# Supplementary material for: Implicit and explicit learning of Bayesian priors differently impacts bias during perceptual decision-making
Source: Sci Rep. 2021 Aug 20;11:16932. doi: 10.1038/s41598-021-95833-7 (PMC8379185; doi:10.1038/s41598-021-95833-7)
Supplement: Supplementary file 1 — Supplementary Information. [file 41598_2021_95833_MOESM1_ESM.pdf]

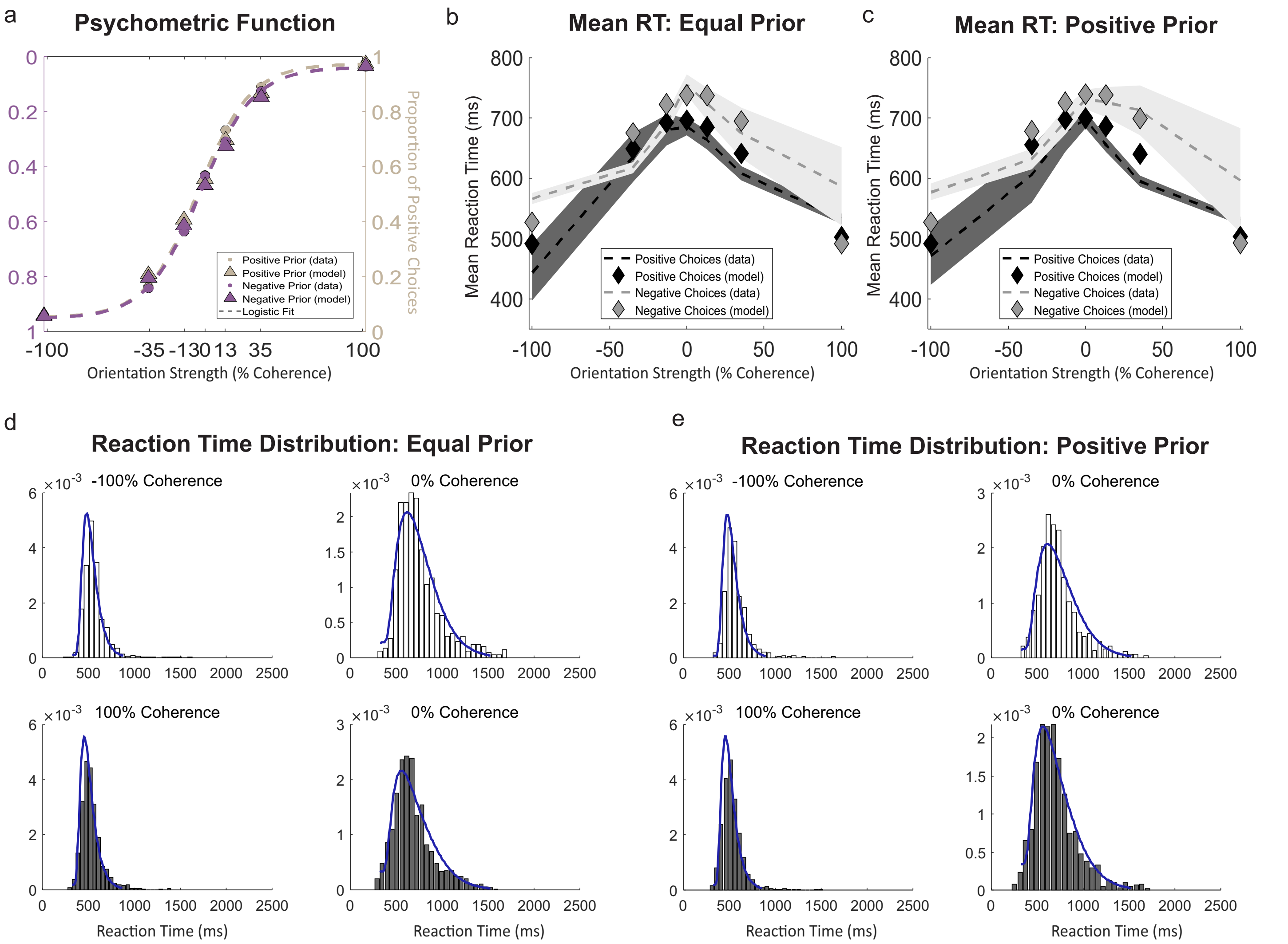

Figure S1. Model fits for choices and reaction times for Implicit condition in Experiment 1.
a. Psychometric function showing proportion of positive choices as a function of coherence for Equal prior condition (*lilac*) and Positive prior condition (*khaki*). Circles shows the data and the triangles show the model predictions and the dashed line shows the maximum likelihood logistic fit to the data.
b. Mean reaction time plot as a function of stimulus coherence for Equal prior condition. The gray color shows the mean RT for negative choices and dark color shows mean RT for positive choices. Dashed line is the mean reaction time and the shaded area shows the 95% confidence interval. The model prediction of mean RT is shown by the diamonds.
c. Same as b. for Positive Prior condition.
d. Reaction Time distributions under Equal prior condition for i) 100% coherence in negative direction and negative choices (gray, upper left), ii) 0% coherence and negative choices (gray, upper right), iii) 100% coherence in positive direction and positive choices (dark, lower left), and iv) 0% coherence and positive choices (dark, lower right). The histogram shows the data and blue traces show the model prediction.
e. Same as d. for Positive prior condition.

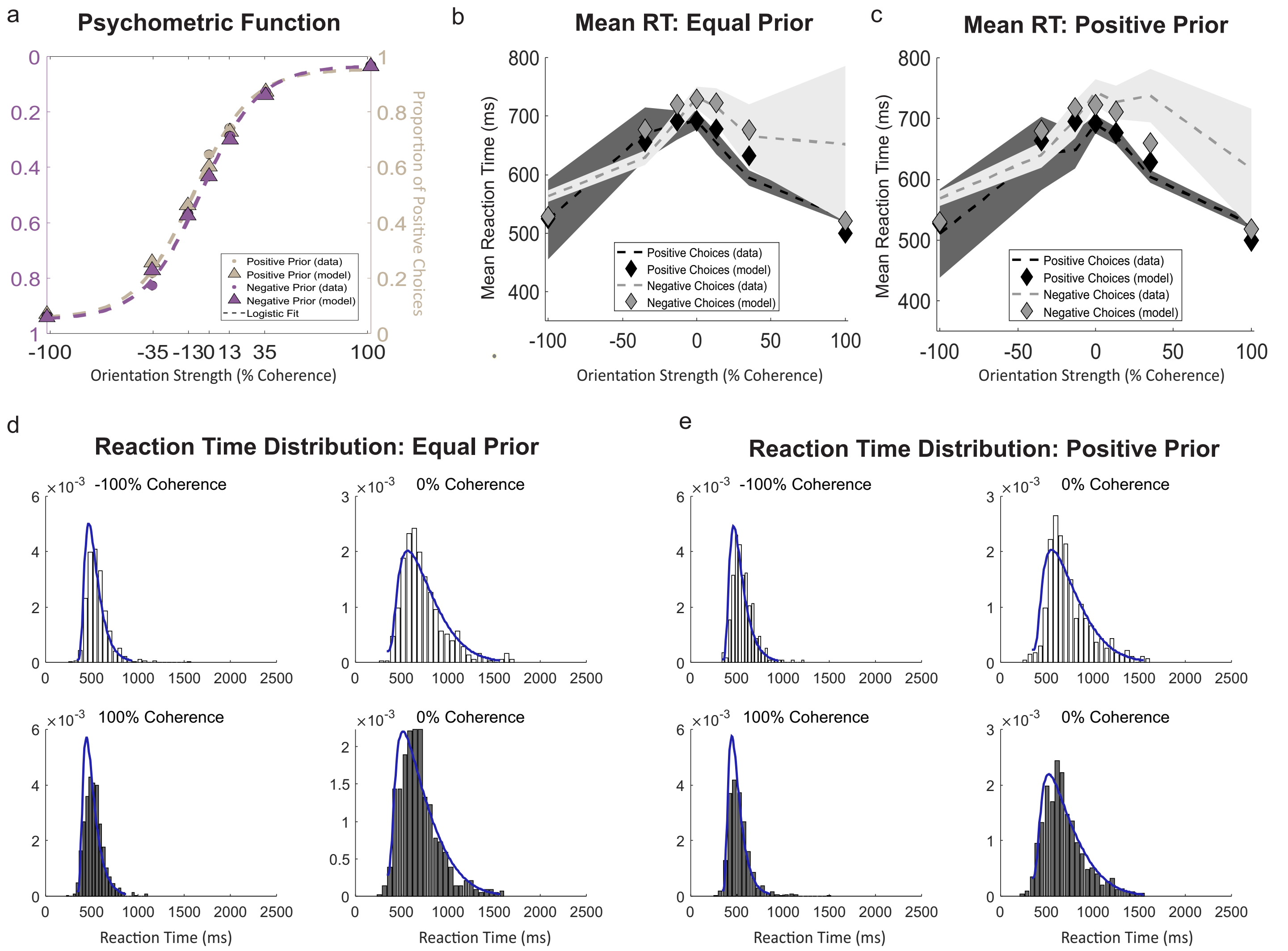

Figure S2. Model fits for choices and reaction times for Partial Explicit condition in Experiment 1.
a. Psychometric function showing proportion of positive choices as a function of coherence for Equal prior condition (*lilac*) and Positive prior condition (*khaki*). Circles shows the data and the triangles show the model predictions and the dashed line shows the maximum likelihood logistic fit to the data.
b. Mean reaction time plot as a function of stimulus coherence for Equal prior condition. The gray color shows the mean RT for negative choices and dark color shows mean RT for positive choices. Dashed line is the mean reaction time and the shaded area shows the 95% confidence interval. The model prediction of mean RT is shown by the diamonds.
c. Same as b. for Positive Prior condition.
d. Reaction Time distributions under Equal prior condition for i) 100% coherence in negative direction and negative choices (gray, upper left), ii) 0% coherence and negative choices (gray, upper right), iii) 100% coherence in positive direction and positive choices (dark, lower left), and iv) 0% coherence and positive choices (dark, lower right). The histogram shows the data and blue traces show the model prediction.
e. Same as d. for Positive prior condition.

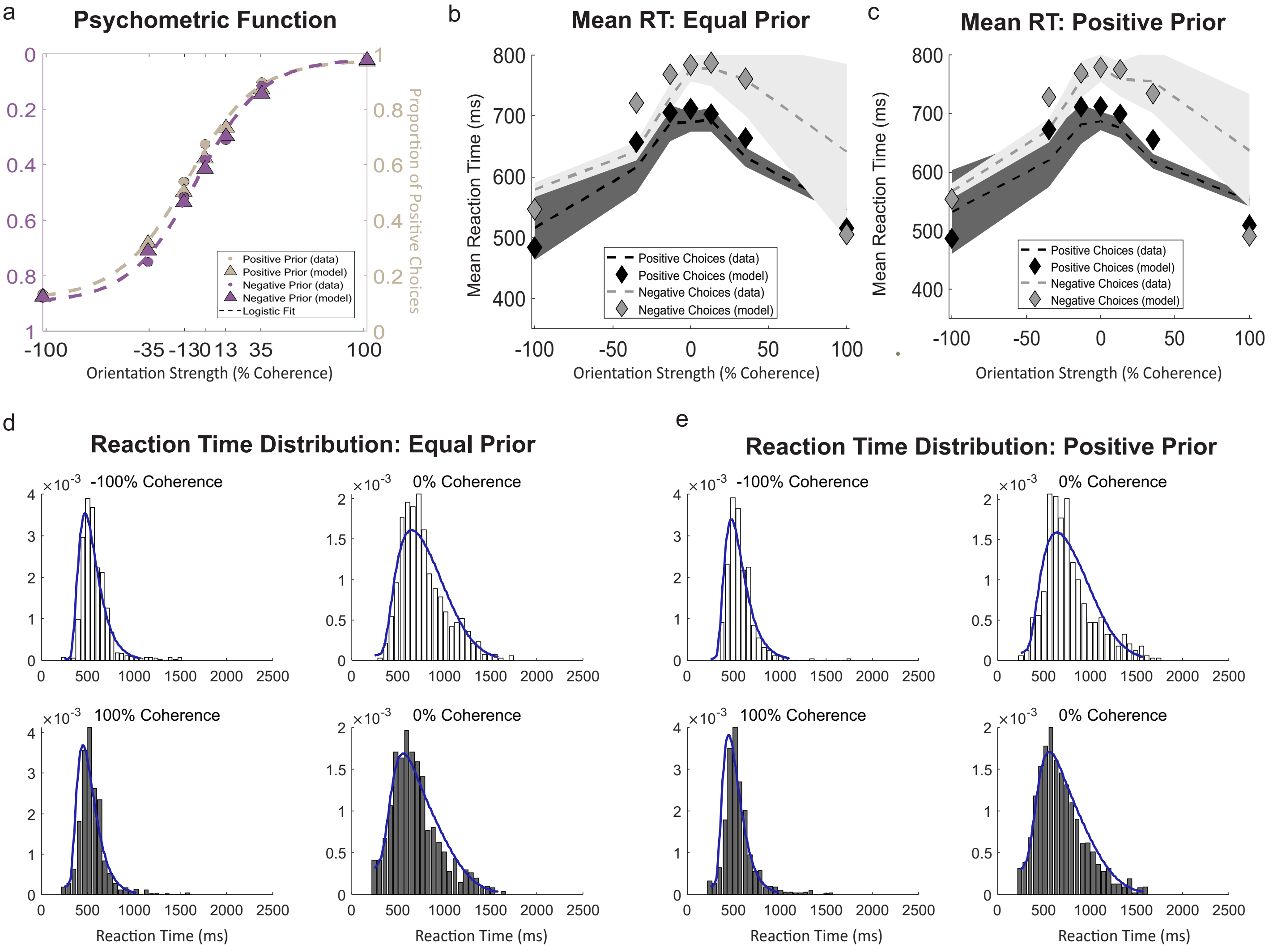

Figure S3. Model fits for choices and reaction times for Explicit condition in Experiment 1.
a. Psychometric function showing proportion of positive choices as a function of coherence for Equal prior condition (*lilac*) and Positive prior condition (*khaki*). Circles shows the data and the triangles show the model predictions and the dashed line shows the maximum likelihood logistic fit to the data.
b. Mean reaction time plot as a function of stimulus coherence for Equal prior condition. The gray color shows the mean RT for negative choices and dark color shows mean RT for positive choices. Dashed line is the mean reaction time and the shaded area shows the 95% confidence interval. The model prediction of mean RT is shown by the diamonds.
c. Same as b. for Positive Prior condition.
d. Reaction Time distributions under Equal prior condition for i) 100% coherence in negative direction and negative choices (gray, upper left), ii) 0% coherence and negative choices (gray, upper right), iii) 100% coherence in positive direction and positive choices (dark, lower left), and iv) 0% coherence and positive choices (dark, lower right). The histogram shows the data and blue traces show the model prediction.
e. Same as d. for Positive prior condition.

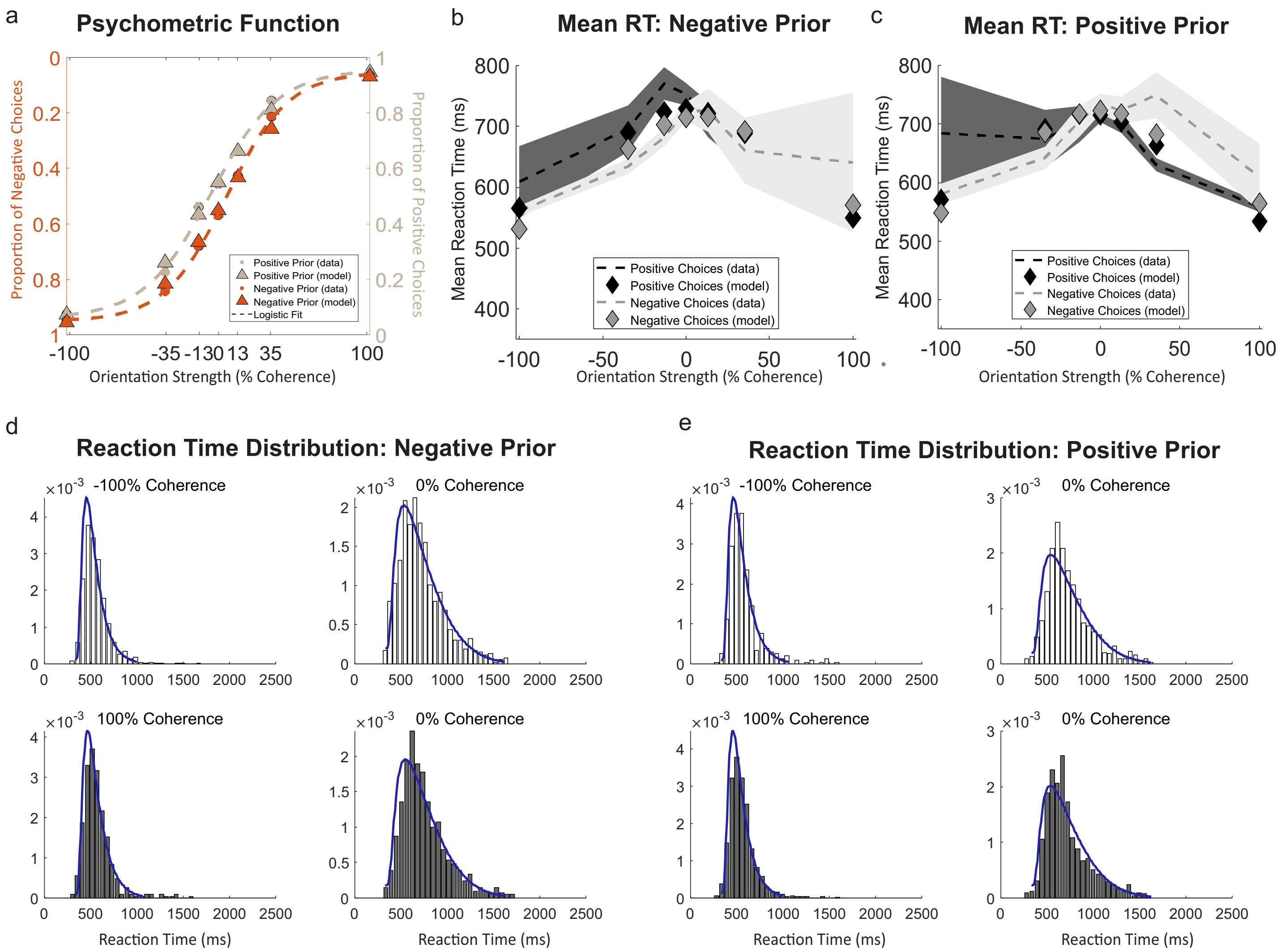

Figure S4. Model fits for choices and reaction times for Implicit condition in Experiment 2.
a. Psychometric function showing proportion of positive choices as a function of coherence for Negative prior condition (orange) and Positive prior condition (*khaki*). Circles shows the data and the triangles show the model predictions and dashed line shows the maximum likelihood logistic fit to the data.
b. Mean reaction time plot as a function of stimulus coherence for the Negative prior condition. The gray color shows the mean RT for negative choices and dark color shows mean RT for positive choices. Dashed line is the mean reaction time and the shaded area shows the 95% confidence interval. The model prediction of mean RT is shown by the diamonds.
c. Same as b. for Positive Prior condition.
d. Reaction time distributions under Negative prior condition for i) 100% coherence in negative direction and negative choices (gray, upper left), ii) 0% coherence and negative choices (gray, upper right), iii) 100% coherence in positive direction and positive choices (dark, lower left), and iv) 0% coherence and positive choices (dark, lower right). The histogram shows the data and blue traces show the model prediction.
e. Same as d. for Positive prior condition.

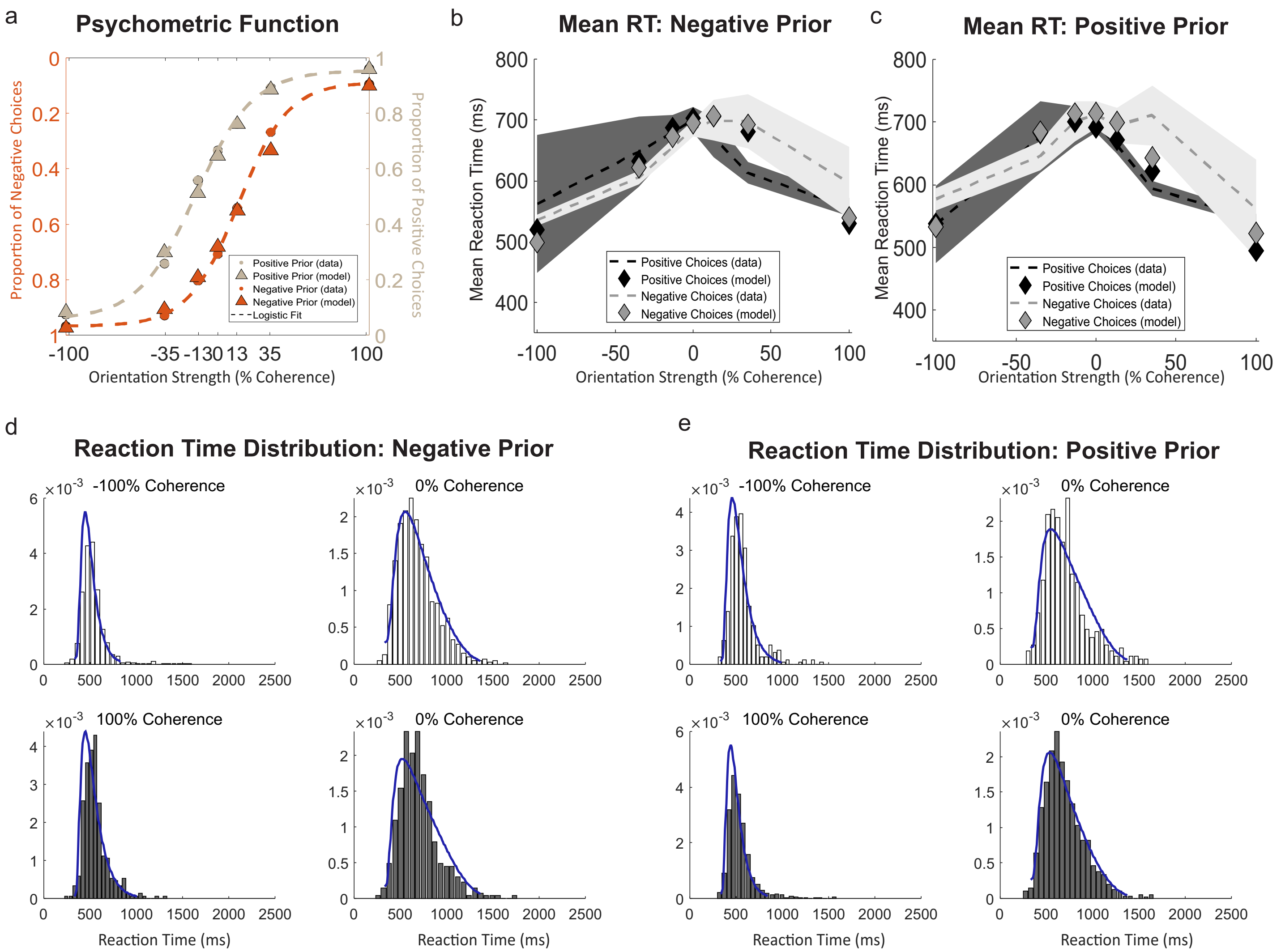

Figure S5. Model fits for choices and reaction times for Explicit condition in Experiment 2.
a. Psychometric function showing proportion of positive choices as a function of coherence for Negative prior condition (orange) and Positive prior condition (*khaki*). Circles shows the data and the triangles show the model predictions and dashed line shows the maximum likelihood logistic fit to the data.
b. Mean reaction time plot as a function of stimulus coherence for the Negative prior condition. The gray color shows the mean RT for negative choices and dark color shows mean RT for positive choices. Dashed line is the mean reaction time and the shaded area shows the 95% confidence interval. The model prediction of mean RT is shown by the diamonds.
c. Same as b. for Positive Prior condition.
d. Reaction time distributions under Negative prior condition for i) 100% coherence in negative direction and negative choices (gray, upper left), ii) 0% coherence and negative choices (gray, upper right), iii) 100% coherence in positive direction and positive choices (dark, lower left), and iv) 0% coherence and positive choices (dark, lower right). The histogram shows the data and blue traces show the model prediction.
e. Same as d. for Positive prior condition.

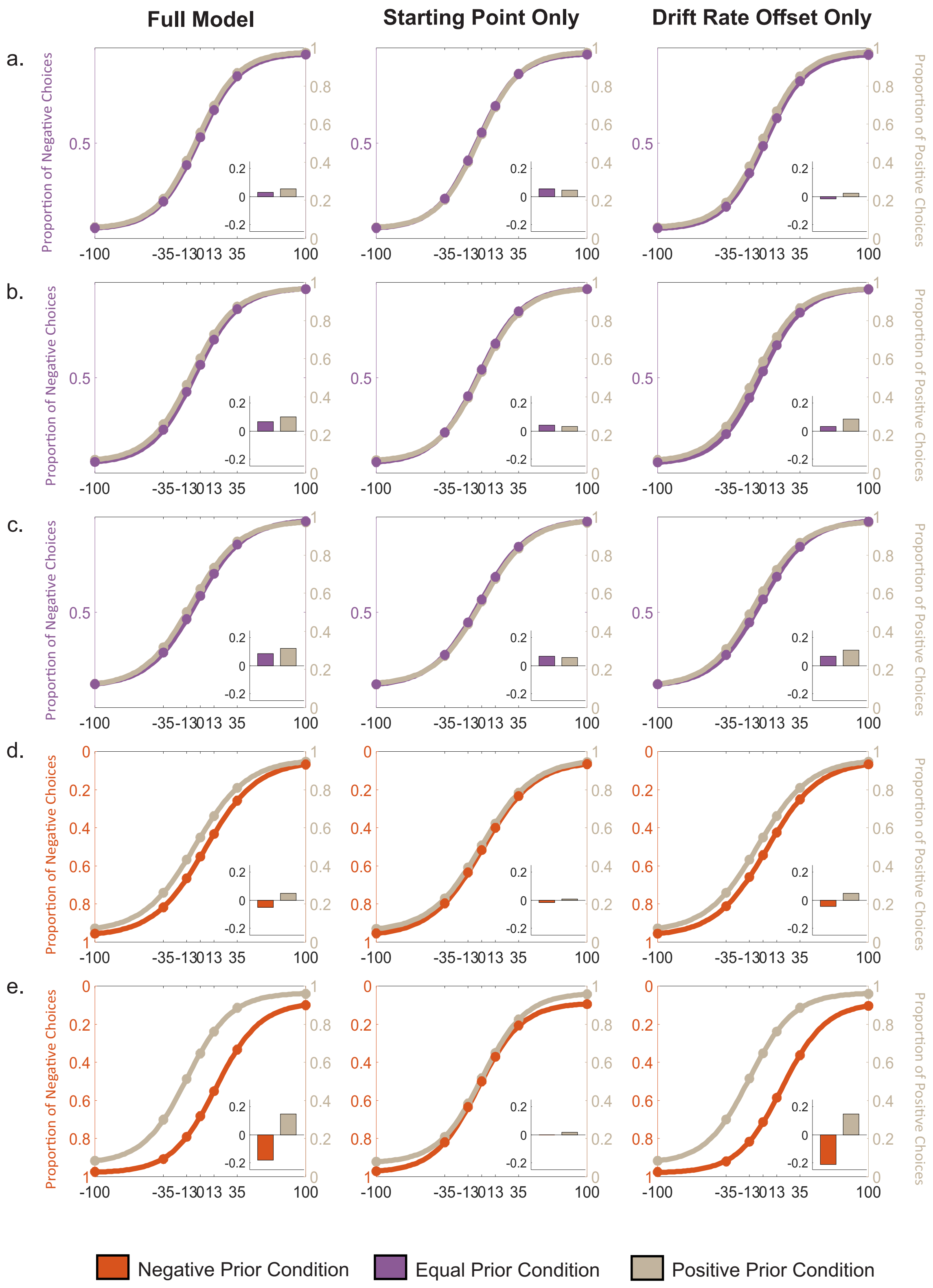

Figure S6: DDM model simulation for experiment 1 (a, b and c) and experiment 2 (d and e). a. Proportion of choices plotted against Glass pattern coherences in Implicit condition during experiment 1. The psychometric function was generated by simulating DDM model with optimized parameters including starting point offset and drift rate offset (first column), only starting point offset (second column) and only drift rate offset (third column). The inset in each figure shows the bias at 0% coherence for respective stimulus feature compared to chance level. b. same as in a for Partial Implicit condition in Experiment 1, c. same as in a for explicit condition in Experiment 1, d. same as in a for Implicit condition in Experiment 2, and e. same as in a for explicit condition in Experiment 2.

Table S1: Model parameters for Experiment 1. Related to Figure 2.

Parameters		Implicit		Partial Explicit		Explicit	
		Estimate	*S.E.*	*Estimate*	*S.E.*	*Estimate*	*S.E.*
**Proportionality factor between coherence and drift rate *k* (1/s·% coh) x 10^{-5}**		3.3650	0.0442	4.5250	0.04841	2.525	0.04707
**Diffusion coefficient (variance/time) (s^{-1})**		$8.8747e^{-4}$	$3.4654e^{-5}$	0.0014	$2.6232e^{-5}$	$7.0002e^{-4}$	$5.0839e^{-5}$
**Scaling parameter for collapsing bounds *s* (s^{-1})**		0.0047	$9.909e^{-5}$	0.0030	2.6076	0.0033	$1.8472e^{-4}$
**Delay parameter for collapsing bounds *d* (ms)**		81.0029	87.3539	1	34.6458	241	34.3183
**Residual time t_0 (ms)**		309	1.8568	326	1.9021	236.9997	2.0751
**Equal Prior Condition**	**Starting point offset**	0.0755	0.007	0.0636	0.0080	0.0318	0.0075
	**Drift-rate offset *O* (s^{-1}) x 10^{-5}**	-7.8201	1.7731	9.9751	2.2828	5.0000	1.5372
	**Probability of positive response**	0.0517	0.0059	0.0519	0.0064	0.1170	0.0050
	**Probability of negative response**	0.0294	0.0063	0.0283	0.0065	0.0155	0.0069
**Positive Prior Condition**	**Starting point offset**	0.0504	0.0070	0.0315	0.0080	0.0215	0.0076
	**Drift-rate offset *O* (s^{-1}) x 10^{-5}**	2.499	1.7689	29.698	2.3150	17.50	1.5122
	**Probability of positive response**	0.0559	0.0058	0.0629	0.0063	0.1166	0.0052
	**Probability of negative response**	0.0202	0.0066	0.0284	0.0063	0.0218	0.0064
**Mean of RT distribution for uninformed decision**		51.3709	18.421	26.3029	24.977	194.1021	10.9023
**SD of RT distribution for uninformed decisions**		196.6445	8.6075	236.7432	10.8925	172.3235	4.8731

Table S2: Model parameters for Experiment 2. Related to Figure 3.

Parameters		Implicit		Explicit	
		Estimate	*S.E.*	*Estimate*	*S.E.*
**Proportionality factor between coherence and drift rate *k* (1/s·% coh) x 10^{-5}**		3.7125	0.0498	4.40	0.049153
**Diffusion coefficient (variance/time) (s^{-1})**		0.0014	$2.837e^{-5}$	0.0014	$2.6619e^{-5}$
**Scaling parameter for collapsing bounds *s* (s^{-1})**		0.0026	$3.2151e^{-4}$	0.0027	$3.7582e^{-4}$
**Delay parameter for collapsing bounds *d* (ms)**		1	38.576	480.996	35.8201
**Residual time t_0 (ms)**		316	2.0365	312	1.877
**Negative Prior Condition**	**Starting point offset**	-0.0159	0.0081	0.0686	0.0084
	**Drift-rate offset *O* (s^{-1}) x 10^{-5}**	-13.781	2.1591	-78.750	2.4168
	**Probability of positive response**	0.0367	0.0065	0.0238	0.0062
	**Probability of negative response**	0.0510	0.0067	0.0883	0.0062
**Positive Prior Condition**	**Starting point offset**	$0.0001e^{-3}$	0.008	-0.0031	0.0084
	**Drift-rate offset *O* (s^{-1}) x 10^{-5}**	17.500	2.2086	55.781	2.3808
	**Probability of positive response**	0.0600	0.0066	0.0754	0.0065
	**Probability of negative response**	0.0419	0.0064	0.0354	0.0059
**Mean of RT distribution for uninformed decision**		128.0017	25.3396	135.2723	17.7876
**SD of RT distribution for uninformed decisions**		247.4817	8.5055	200.3777	7.4011

Table S3: Model comparisons between model with only start point offset, model with only drift-rate offset, and model with both starting point and drift-rate offset. Related to Figure 2 and 3

BIC scores		Only starting point offsets	Only drift-rate offsets	Both types of offsets
**75-50 Experiment**	**Implicit**	67193	67255	66982
	**Partial Explicit**	68162	68034	67892
	**Explicit**	71179	71056	70695
**75-25 Experiment**	**Implicit**	70915	70898	70679
	**Explicit**	68296	68084	68136
